# Alterations and Associations Between Magnetic Susceptibility of the Basal Ganglia and Diffusion Properties in Alzheimer’s Disease

**DOI:** 10.3389/fnins.2021.616163

**Published:** 2021-02-16

**Authors:** Xiuxiu Liu, Lei Du, Bing Zhang, Zifang Zhao, Wenwen Gao, Bing Liu, Jian Liu, Yue Chen, Yige Wang, Hongwei Yu, Guolin Ma

**Affiliations:** ^1^Department of Radiology, China-Japan Friendship Hospital, Beijing, China; ^2^Graduate School of Peking Union Medical College, Beijing, China; ^3^Department of Radiology, The Affiliated Drum Tower Hospital of Nanjing University Medical School, Nanjing, China; ^4^Department of Anesthesiology, Peking University First Hospital, Beijing, China; ^5^Department of Ultrasound Diagnosis, China-Japan Friendship Hospital, Beijing, China; ^6^Institute of Brain Science, Nanjing University, Nanjing, China

**Keywords:** diffusion tensor imaging, quantitative susceptibility mapping, basal ganglia, Alzheimer’s disease, magnetic susceptibility, tract-based spatial statistics

## Abstract

This study adopted diffusion tensor imaging to detect alterations in the diffusion parameters of the white matter fiber in Alzheimer’s disease (AD) and used quantitative susceptibility mapping to detect changes in magnetic susceptibility. However, whether the changes of susceptibility values due to excessive iron in the basal ganglia have correlations with the alterations of the diffusion properties of the white matter in patients with AD are still unknown. We aim to investigate the correlations among magnetic susceptibility values of the basal ganglia, diffusion indexes of the white matter, and cognitive function in patients with AD. Thirty patients with AD and nineteen healthy controls (HCs) were recruited. Diffusion indexes of the whole brain were detected using tract-based spatial statistics. The caudate nucleus, putamen, and globus pallidus were selected as regions of interest, and their magnetic susceptibility values were measured. Compared with HCs, patients with AD showed that there were significantly increased axial diffusivity (AxD) in the internal capsule, superior corona radiata (SCR), and right anterior corona radiata (ACR); increased radial diffusivity (RD) in the right anterior limb of the internal capsule, ACR, and genu of the corpus callosum (GCC); and decreased fractional anisotropy (FA) in the right ACR and GCC. The alterations of RD values, FA values, and susceptibility values of the right caudate nucleus in patients with AD were correlated with cognitive scores. Besides, AxD values in the right internal capsule, ACR, and SCR were positively correlated with the magnetic susceptibility values of the right caudate nucleus in patients with AD. Our findings revealed that the magnetic susceptibility of the caudate nucleus may be an MRI-based biomarker of the cognitive dysfunction of AD and abnormal excessive iron distribution in the basal ganglia had adverse effects on the diffusion properties of the white matter.

## Introduction

Alzheimer’s disease (AD) is recognized as a degenerative disease characterized by cognitive dysfunction and memory disorder, and the number of patients with AD is in the first place in senile dementia (Zhong et al., 2004; Arfanakis et al., 2007; Chen et al., 2007). From 2000 to 2017, the number of deaths from AD increased by 45% (Gaugler et al., 2019). Until now, people with AD have suffered because there is no way to stop the progression of AD, so it is critical to find early pathological changes and brain imaging changes for early diagnosis and early treatment. Some studies have shown that amyloid-β (Aβ) plaque, phosphorylated tau protein, and pathological iron deposition in the brain cause damage to the neurons and axons (Hardy, 2006; Pena et al., 2006; Takahashi et al., 2017).

Diffusion tensor imaging (DTI) has recently emerged as a non-invasive and effective neuroimaging method that provides valuable imaging clues to detect changes in the diffusion function of the fiber fasciculus in terms of neuropsychiatric diseases. Axial diffusivity (AxD), radial diffusivity (RD), mean diffusivity (MD), and fractional anisotropy (FA) are the common and representative DTI metrics (Alexander et al., 2007; Amlien and Fjell, 2014; Alves et al., 2015). AxD indicates the velocity of water diffusion along the longitudinal axis, and FA can measure the degree of directionality of water diffusion. Previous DTI studies have explored that white matter fiber alterations are widespread in AD. Generally, the alterations of the white matter in AD exist in the corpus callosum, cingulum bundle, internal capsule, fornix, as well as prefrontal lobe, posterior parietal, and medial frontal regions of the white matter (Sun et al., 2004; Naggara et al., 2006; Mielke et al., 2009; Teipel et al., 2012; Madhavan et al., 2016; Araque Caballero et al., 2018).

Quantitative susceptibility mapping (QSM) is a non-invasive MRI technology that enables to quantify the magnetic susceptibility properties of the tissues *in vivo* (Schweser et al., 2012), and the susceptibility value can be used to reflect the iron level of the brain tissue. Several studies have shown that excessive iron deposition was found in some degenerative diseases, such as AD and Parkinson’s disease (PD) (Mitsumori et al., 2009; Oshiro et al., 2011). Furthermore, studies about AD in *in vivo*, *in vitro*, postmortem, and animal models have found that the magnetic susceptibility values were increased in the basal ganglia, such as the caudate nucleus and the putamen (Acosta-Cabronero et al., 2013; Hwang et al., 2016; Moon et al., 2016).

Although previous studies have explored the brain iron overload and diffusion parameter changes, whether the changes of susceptibility values due to excessive iron in the basal ganglia have correlations with the alterations of the diffusion properties of the white matter of patients with AD are still unknown. This study aimed to detect the diffusion index changes of the white matter fibers using the tract-based spatial statistics (TBSS) and explored the magnetic susceptibility changes of the basal ganglia. We then examined the relationships between iron deposition and the changes of white matter integrity in AD patients.

## Materials and Methods

### Subjects

The ethics committee of China–Japan Friendship Hospital has approved this study, and the study obtained informed consent from all subjects. Thirty patients with AD and twenty healthy controls (HCs) were recruited in this study. AD patients were recruited when they visited the Department of Neurology of China–Japan Friendship Hospital in November 2015 and March 2019. Inclusion criteria of patients with AD were as follows: (a) conforming to the diagnostic criteria of the National Institute of Neurological and Communicative Disorders and Stroke and the Alzheimer’s Disease and Related Disorders Association (NINCDS-ADRDA) (Acosta-Cabronero et al., 2013; Du et al., 2018), (b) no abnormal brain changes in conventional MRI examination, (c) right-handed, and (d) the MRI images are complete and have no artifacts. Exclusion criteria of patients with AD were as follows: (a) brain organic lesions; (b) neuropsychiatric diseases; (c) alcohol and drug dependence; (d) severe history of heart, lung, liver, and kidney diseases; (e) metabolic diseases, such as hypothyroidism and vitamin B12 deficiency; and (f) MRI contraindications. HCs were recruited from the local communities. Inclusion conditions of HCs were as follows: (a) no psychoses and neurological diseases, (b) no abnormal brain changes in conventional MRI examination, (c) Mini-Mental State Examination (MMSE) scores were between 26 and 30, (d) right-handed, and (d) the MR images are complete and have no artifacts. One HC was excluded because of incomplete images. The total number of all subjects is 49, which consists of 30 patients with AD and 19 HCs. The MMSE scale and Montreal Cognitive Assessment (MoCA) scale were used to assess the cognitive performance of all patients. All participants received conventional MRI, DTI, and QSM scanning.

### MRI Scan Acquisition

MRI examinations consisting of DTI and QSM were executed on a 3.0 T device using eight-channel head coils (Discovery MR750 scanner; GE Medical Systems, United States). Research sequences included a spin-echo echo-planar sequence (EPI) to acquire the DTI images with 64 encoding directions (b = 1,000 s/mm^2^) and eight b0 reference images (b = 0 s/mm^2^) [repetition time (TR) = 8,028 ms, echo time (TE) = 81.8 ms, flip angle (FA) = 90°, slice thickness = 2 mm, matrix = 120 × 120, and field of view (FOV) = 240 × 240 mm^2^] and a 3D gradient echo (GRE) sequence to acquire QSM images (TE = 3.2 ms, TR = 22.9 ms, FA = 12°, slice thickness = 1 mm, bandwidth = 62.5 Hz/pixel, FOV = 256 × 256 mm^2^, matrix size = 256 × 256).

### Image Preprocessing

Briefly, DTI preprocessing included images format conversion using the dcm2niix tool, head motion eddy correction, gradient direction correction using the FMRIB’s Diffusion Toolbox (FDT), acquisition of brain mask using the Brain Extraction Tool (BET), and diffusion tensor calculation to output AxD, MD, FA, and RD (Lin et al., 2020). The above procedures used the FSL software^1^ running in Linux.

QSM reconstruction included loading magnitude/phase images, creating brain mask based on the magnitude images, phase unwrapping based on Laplacian algorithm, background phase removing, using the streaking artifact reduction method for QSM (STAR-QSM) to reconstruct QSM images, and region of interest (ROI) drawing were implemented using the STI Suite software^2^ in Matlab 2013b (Li et al., 2011; Schweser et al., 2011; Wei et al., 2015).

### TBSS Processing

After diffusion tensor images were preprocessed, voxel-wise analysis was performed using the TBSS toolbox. Individual FA images were mapped onto the FMRIB58 FA template, performed the non-linear registration to transform into Montreal Neurological Institute 152 (MNI152) standard space, and skeletonized using standard parameters (Smith et al., 2006). The mean FA images and the mean FA skeleton maps were generated, taking a threshold of 0.2 for the mean FA skeleton. Furthermore, individual FA images, AxD images, RD images, and MD images were mapped onto the mean FA skeleton, respectively. The code tbss_fill was used for results expansion, which can visualize the manifestation of realistic analysis easily. FSLview was adopted to view results. Some auxiliary commands, such as cluster, atlasquery, and fslmeants, were used. The atlasquery command was used to output information about the white matter fibers in clusters with significant differences. Anatomical atlas consisting of JHU white-matter tractography atlas and JHU ICBM-DTI-81 white-matter labels could identify various fiber tracts (Wakana et al., 2004; Hua et al., 2008). Afterward, the cluster program was used to acquire the statistically significant clusters information about AxD, RD, and FA (Chen et al., 2018). The fslmeants command is used to extract the mean values of the diffusion indexes of the significant difference clusters for other analysis.

### Basal Ganglia Segmentation and Measurement

**FIGURE 1:**
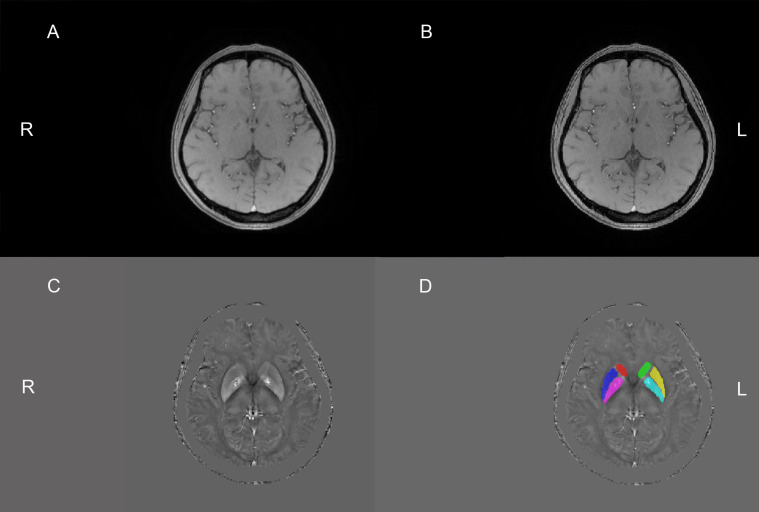
The above four pictures are the same slice of the same patient. The raw and unprocessed DICOM image **(A)** obtained from the MRI machine. Sensitive information, such as the patient’s name, check number, and date of birth, has been covered with black. The magnitude image **(B)** and QSM image **(C)** were obtained by the post-processing of the STI Suite software, respectively. This image **(D)** was obtained after drawing the regions of interest (ROIs) based on the C image. Six ROIs were drawn manually on QSM images. Red, dark blue, purple, green, yellow, and light blue are the right caudate nucleus, right putamen, right globus pallidus, left caudate nucleus, left putamen, and left globus pallidus, respectively. R, right; L, left.

Some raw and unprocessed DICOM images, post-processed magnitude images, and QSM images of the same slices from the same patient’s brain can be seen in Supplementary Figure 1. After acquiring the final QSM images, we selected the basal ganglia as the ROI, and it was further divided into six ROIs: bilateral caudate nucleus, bilateral putamen, and bilateral globus pallidus as shown in Figure 1. The six ROIs are demarcated on the QSM axial images. Each ROI was manually segmented and measured the susceptibility values in three consecutive layers on axial QSM images by two radiologists to improve measurement accuracy and verify measurement consistency. They drew and measured ROIs independently and knew nothing about the subjects. The unit of susceptibility value was the international standard unit—parts per million (ppm).

### Statistical Analysis

Statistical analysis was executed using the SPSS software package (IBM SPSS 26.0). The Shapiro–Wilk normality test was used to test data for normality. All variables were normally distributed except the magnetic susceptibility values of the bilateral caudate nucleus and right globus pallidus in the AD group. Two-sample *t*-test or non-parameter testing of two independent samples was used for intergroup comparison. Gender was compared using the chi-square (χ^2^) test. The intraclass correlation coefficient (ICC) was calculated to evaluate the concordance between the two radiologists’ measurements (Oppo et al., 1998; Du et al., 2018). Group differences in FA, MD, RD, and AxD were analyzed running the FSL randomise program with permutation tests (5,000 permutations) for multiple comparisons adopting the threshold-free cluster enhancement (TFCE) method (Nichols and Holmes, 2002; Winkler et al., 2014; Goodrich-Hunsaker et al., 2018). We chose gender, age, and educational years as covariates in the regression analysis (Sampedro et al., 2020). P value less than 0.05 was considered statistically different. Partial correlation analysis controlling age and gender was used for correlation analysis, and false discovery rate (FDR) was used for multiple comparison correction. Combining the FA values with the susceptibility values of the right caudate nucleus, the MMSE scores were used for multiple linear regression analysis to discover which imaging parameters contribute more to cognitive decline.

## Results

### Subjects’ Characteristics

Participants’ demographic characteristics and the results of cognitive scales are presented in Table 1. MMSE and MoCA scores were used to assess the cognitive function of AD patients. The MMSE test revealed lower scores in AD patients than in HCs (*P* < 0.001). There was no significant difference in gender, age, and educational years between the AD group and the HC group.

**TABLE 1:** Demographic and clinical characteristics.

Group	AD	Healthy controls	*P*
Number	30	19	–
Male/female	8/22	5/14	0.978
Age (year)	68.37 ± 6.734	66.68 ± 8.564	0.447
Education (year)	11.03 ± 3.917	9.63 ± 3.961	0.230
MMSE	19.80 ± 3.925	28.00 ± 1.856	0.000
MoCA	16.77 ± 3.857	Nm	–

*MMSE, Mini-Mental State Examination; MoCA, Montreal Cognitive Assessment; Nm, not measured.*

Two experienced radiologists drew the ROIs and measured their magnetic susceptibility values on QSM images. ICC was calculated to show consistency between two observers. The susceptibility values measured by two radiologists were consistent, as shown in Supplementary Table 1.

### Mapping Intergroup Differences of TBSS Analysis

**FIGURE 2:**
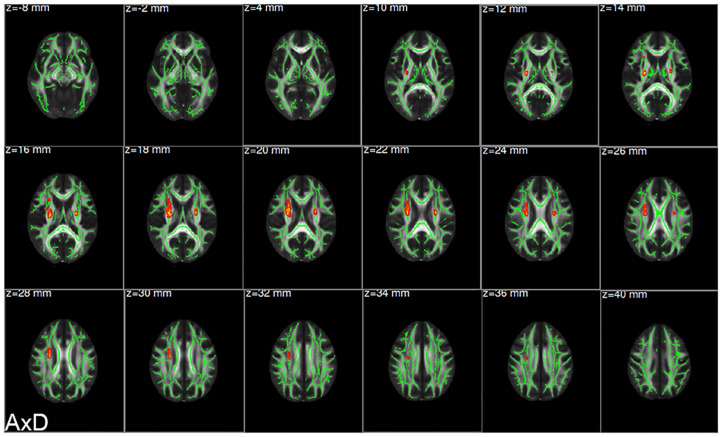
Clusters showing significant difference in axial diffusivity (AxD) values between the AD group and the HC group. The red clusters were attached to the axial images of the mean FA skeleton (green), showing significantly increased AxD values in the AD group compared with the HC group (AD > HC; FWE corrected *P* < 0.05). FWE, family wise error; AD, Alzheimer’s disease; HC, healthy controls.

**FIGURE 3:**
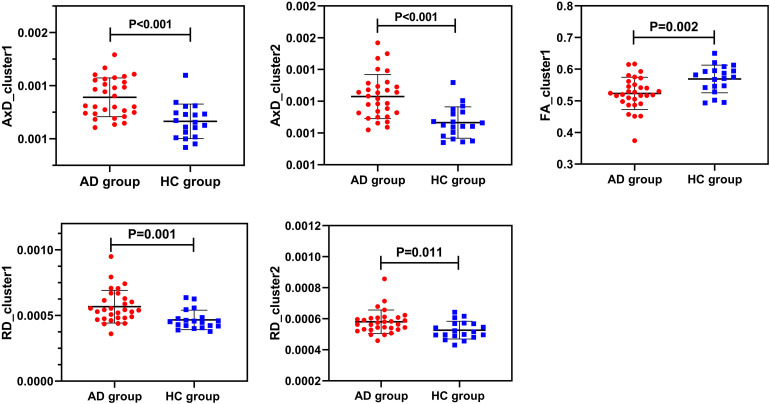
Intergroup comparisons of AxD values, RD values, and FA values between the AD group (red) and the HC group (blue). AxD values, RD values, and FA values of clusters were statistically different between the two groups before FWE correction. AxD, axial diffusivity; RD, radial diffusivity; FA, fractional anisotropy; AD, Alzheimer’s disease; HC, healthy controls.

There were statistically significant differences in AxD values between the AD group and the HC group after family wise error (FWE) correction (*P* < 0.05) (Figure 2). The corresponding statistically significant clusters were displayed in Table 2. The AD group had significantly higher AxD values than the HC group in two clusters including the bilateral superior corona radiata (SCR), bilateral posterior limb of the internal capsule (PLIC), bilateral anterior limb of the internal capsule (ALIC), and right anterior corona radiata (ACR). Higher RD values could be found in the right ACR, right ALIC, and genu of the corpus callosum (GCC) in AD patients. However, there was no significant difference between the AD group and the HC group after FWE correction, which was displayed in Supplementary Figure 2 and Supplementary Table 2. Lower FA values were observed in the right ACR and GCC in the AD group than in the HC group, but no significant difference after FWE multiple comparison correction, which was shown in Supplementary Figure 3 and Supplementary Table 3. The regions with statistically different MD values did not exist after FWE multiple comparison correction. The mean AxD, RD, and FA values of every cluster were extracted and compared between the AD group and the HC group, and the corresponding statistically significant results without FWE multiple comparison correction were shown in Figure 3.

**TABLE 2:** Clusters showed significant differences in AxD values.

Cluster ID	Cluster size	P-FWE	MNI coordinates			Tracts in clusters
			X	Y	z	
**AD > HC**						
Cluster 1	60	0.048	114	121	91	Superior corona radiata (L)
						Posterior limb of the internal capsule (L)
						Anterior limb of the internal capsule (L)
Cluster 2	384	0.034	65	114	86	Superior corona radiata (R)
						Posterior limb of the internal capsule (R)
						Anterior corona radiata (R)
						Anterior limb of the internal capsule (R)

*The MNI coordinate refers to the coordinate with the largest 1 - P value. AxD, axial diffusivity; AD, Alzheimer’s disease; HC, healthy control; FWE, family wise error; MNI, Montreal Neurological Institute; R, right; L, left.*

### Comparisons of Susceptibility Values in the Basal Ganglia Between Groups

**FIGURE 4:**
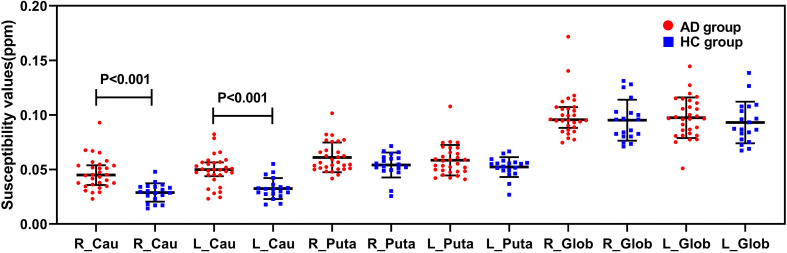
Comparisons of susceptibility values in the basal ganglia between the AD group (red) and the HC group (blue). The susceptibility values of the bilateral caudate nucleus were significantly higher in the AD group than in the HC group (*P* < 0.001). R, right; L, left; Cau, caudate nucleus; Puta, putamen; Glob, globus pallidus.

Figure 4 showed the results about the comparisons of the susceptibility values in the basal ganglia between the AD group and the HC group. Compared with the HC group, the susceptibility values of the bilateral caudate nucleus were significantly increased in the AD group (*P* < 0.001).

### Correlations Between AxD Values, RD Values, FA Values, and Cognitive Scales

**FIGURE 5:**
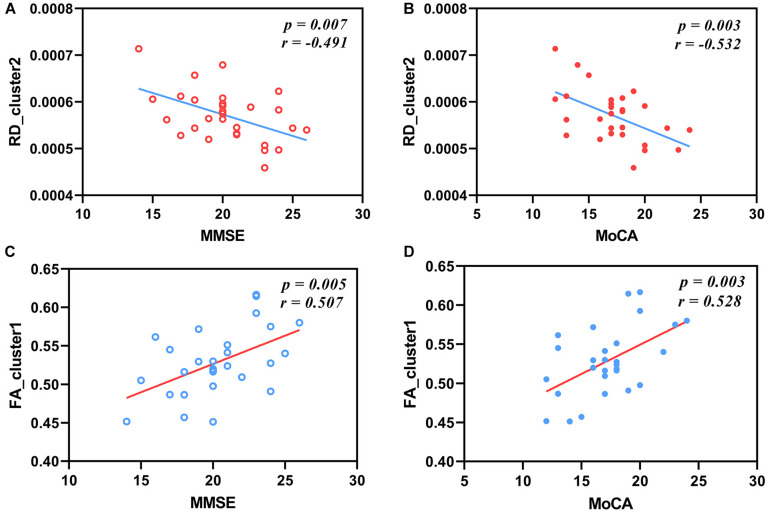
Correlations between RD values, FA values, and cognitive scales in patients with AD. The mean RD values of cluster 2 have negative correlations with MMSE scores **(A)** and MoCA scores **(B)** after FDR correction (*r* = −0.491, *P* = 0.007; *r* = −0.532, *P* = 0.003). The mean FA values of cluster 1 have positive correlations with MMSE scores **(C)** and MoCA scores **(D)** after FDR correction (*r* = 0.507, *P* = 0.005; *r* = 0.528, *P* = 0.003). RD, radial diffusivity; FA, fractional anisotropy; MMSE, Mini-Mental State Examination; MoCA, Montreal Cognitive Assessment.

We found that the MMSE and MoCA scores of one patient were extremely low, so we removed the MMSE and MoCA scores of this patient in order to acquire reliable analysis results. The results of partial correlation analysis between the AxD values, RD values, and FA values and the MoCA scores and MMSE scores in patients with AD were shown in Figure 5. After FDR correction, the mean RD values of cluster 2 including the right ACR and GCC had significant negative correlations with the MMSE scores and the MoCA scores (*r* = −0.491, *P* = 0.007; *r* = −0.532, *P* = 0.003; Figures 5A,B). Significant positive correlations between the mean FA values and the MMSE scores or the MoCA scores were observed in Figures 5C,D (*r* = 0.507, *P* = 0.005; *r* = 0.528, *P* = 0.003). There were no significant correlations between AxD values and cognitive scores.

### Correlations Between Susceptibility Values of ROIs and Cognitive Scales

**FIGURE 6:**
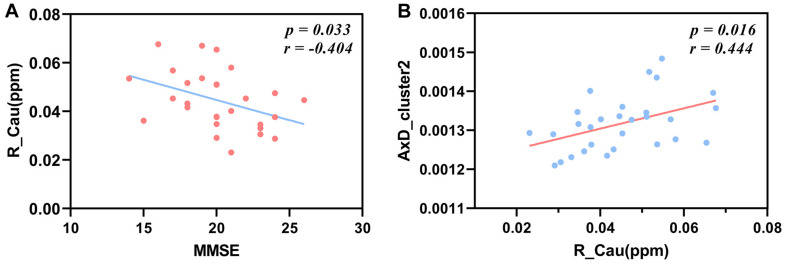
Correlations between the susceptibility values of the right caudate nucleus and MMSE scores, between the susceptibility values of the right caudate nucleus and the AxD values of cluster 2 in patients with AD. The susceptibility values of the right caudate nucleus have a negative correlation with MMSE scores after FDR correction **(A)** (*r* = −0.404, *P* = 0.033). The mean AxD values of cluster 2 have a positive correlation with the susceptibility values of the right caudate nucleus after FDR correction **(B)** (*r* = 0.444, *P* = 0.016). MMSE, Mini-Mental State Examination; AxD, axial diffusivity; R_Cau, right caudate nucleus.

We found that the susceptibility value of the right caudate nucleus of one patient was extremely high and it belonged to an abnormal outlier, so we removed it in order to acquire reliable analysis results. After FDR correction, the susceptibility values of the right caudate nucleus have a significant negative correlation with the MMSE scores in Alzheimer’s patients (*r* = −0.404, *P* = 0.033; Figure 6A).

### Correlations Between Diffusion Parameters and Susceptibility Values of the Right Caudate Nucleus

The mean AxD values of cluster 2 including the right ALIC, right PLIC, right ACR, and right SCR were positively correlated with the susceptibility values of the right caudate in patients with AD after FDR correction (*r* = 0.444, *P* = 0.016; Figure 6B).

### Multiple Linear Regression Analysis

In multiple linear regression analysis, the FA values and the susceptibility values of the right caudate nucleus were independent predictors, and the MMSE scores were used to assess the cognitive function of patients with AD. We combined the FA values with the susceptibility values of the right caudate nucleus to find out which contributed more to the cognitive decline in patients with AD. It was shown in Table 3 that both the FA values and the susceptibility values of the right caudate nucleus contributed to cognition decline, but the latter contributed more.

**TABLE 3:** Multiple linear regression results for different predictors.

Predictors	Coefficients	Standard error	*P* value	VIF	R^2^ (%)
Intercept	7.277	5.864	0.002		39
FA_cluster1	31.681	10.269	0.005	1.009	
R_Cau	−87.513	38.070	0.030	1.009	

*VIF, variance inflation factor.*

## Discussion

This study mainly found increased AxD values and RD values and decreased FA values of patients with AD compared with HCs. The susceptibility values of the bilateral caudate nucleus were increased in patients with AD. The increased AxD values in patients with AD were discovered in the right ACR, bilateral SCR, PLIC, and ALIC. Higher RD values were discovered in the right ACR, ALIC, and GCC in patients with AD. Similarly, decreased FA values were observed in the right ACR and GCC. In addition, RD values and FA values were significantly correlated with cognitive scores, and the susceptibility values of the right caudate nucleus had a significant negative correlation with MMSE scores. It is worth noting that AxD of cluster 2 including the right SCR, ACR, ALIC, and PLIC was positively correlated with the magnetic susceptibility values of the right caudate nucleus.

Our findings found that patients with AD showed higher AxD values in the right ACR, bilateral SCR, ALIC, and PLIC and higher RD values in the right ACR, ALIC, and GCC than HCs. In addition, RD values had significantly negative correlations with the MMSE scores and the MoCA scores. Several previous articles summarized the white matter fiber bundles related to important physiological function, such as the internal capsule and the corpus callosum (Medina and Gaviria, 2008; Lo Buono et al., 2020). The internal capsule, corpus callosum, and corona radiate have complex anatomic connectivity that supports cognitive, sensory, and motor systems in the cortex (Gage and Baars, 2018). A good example is the presence of frontal bridge bundles and prethalamic radiation in the ALIC, if the ALIC is damaged, it may cause memory loss, anxiety, inattention, and so on. Our findings were consistent with some previous studies (Pievani et al., 2010; Chitra et al., 2017; Mayo et al., 2017, 2019; Bigham et al., 2020). Increased AxD values are relevant to axonal injury and Wallerian degeneration in patients. Similarly, alterations in AxD may imply axonal damage, and RD is likely to reflect changes in myelination (Alm and Bakker, 2019). Another explanation is that decreased tissue density can increase water diffusivity, but the basic fiber directional structure remains the same in patients with AD (Englund, 1998; Bronge et al., 2002). Besides, AxD is more likely affected by myelination, axonal diameters, and axonal packing (Song et al., 2003).

Similarly, we also observed that decreased FA values existed in the right ACR and GCC of patients with AD. Furthermore, the FA values and the cognitive scores including MMSE and MoCA were positively correlated. Our findings are consistent with the findings of previous studies; both found that the FA values of patients with AD are reduced (Naggara et al., 2006; Ukmar et al., 2008; Liu et al., 2011; Brueggen et al., 2019). Past studies have also shown that cognitive function is associated with diffusion indexes (Vasconcelos et al., 2009; Ling et al., 2011; Mayo et al., 2019), suggesting that white matter damage adversely affects cognitive function. FA reflects the dispersion direction of the white matter, the regular and organized white matter has higher FA values, and if the structure of the white matter becomes disordered, FA values will decrease. Therefore, FA stands for the integrity of the white matter (Stebbins and Murphy, 2009). The decrease of FA in patients with cognitive impairment may be related to the histopathological features (Parente et al., 2008). The water content of the white matter increases with the decrease of the myelin component under various pathological states (Ringman et al., 2007). The pathological basis of AD is β-amyloid deposition and neurofibrillary tangles, which can damage axonal microstructure, myelin integrity, and axonal transport (Englund, 1998; Ukmar et al., 2008). Changes in the microstructure of the white matter can affect the diffusion motion of water molecules in the brain tissues, which can be reflected and measured by using the diffusion parameters of DTI (Hanyu et al., 1997; Horsfield and Jones, 2002).

As a diffusion imaging technique, DTI is highly sensitive to changes in the white matter fiber tracts and is a valuable method for studying neurodegenerative diseases. Unlike AD, PD is a chronic progressive neurodegenerative disease. The main lesions are in the substantia nigra and striatum pathways. Bradykinesia, resting tremor, myotonia, and postural instability are the four basic manifestations of the disease. Meta-analysis studies of DTI showed decreased FA and/or increased MD in the substantia nigra, corpus callosum, frontal lobe, cingulate, and temporal lobe of patients with PD (Cochrane and Ebmeier, 2013; Deng et al., 2018). Amyotrophic lateral sclerosis (ALS) is a progressive neurodegenerative disease involving the upper and lower motor neurons, characterized by progressive weakness of the limbs, respiratory muscles, and medulla. Reviews of DTI indicated decreased FA and/or increased RD, MD, and AxD in the corticospinal tract, PLIC, corpus callosum, and frontal white matter in patients with ALS (Filippini et al., 2010; Li et al., 2012). Huntington’s disease is an autosomal dominant neurodegenerative disease. Huntington protein accumulates in the brain and affects brain structure and function. Dyskinesia, mental disorder, and dementia are the three main characteristics of the disease. Meta-analysis study of DTI showed increased FA in the caudate, putamen, and globus pallidus, increased MD in the putamen and thalamus, decreased FA in the corpus callosum, increased RD and AD in the corpus callosum of patients with Huntington’s disease (Liu et al., 2016). There are some overlapping results in DTI studies of different neurodegenerative diseases, which may be related to the similar symptoms of these diseases, and the differences in DTI results may be related to specific structural abnormalities in specific degenerative diseases.

It is known that the susceptibility value can be used to reflect the iron level of the brain tissue. Our findings showed that the susceptibility values of the bilateral caudate nucleus increased in patients with AD and the magnetic susceptibility of the right caudate nucleus was negatively correlated with MMSE scores. The association can be supported by another study that ferritin levels of the cerebrospinal fluid have positive connection with cognitive performance (Ayton et al., 2015). Our previous work has found that the increased susceptibility values of the left caudate have decreased cognitive scores, once again proving that higher susceptibility values in the caudate nucleus, a proxy for tissue iron, can predict the cognitive decline. In multiple linear regression analysis, we discovered that both the FA values and the susceptibility values of the right caudate nucleus contributed to cognition decline, but the latter contributed more, which illustrates that the susceptibility values of the caudate nucleus influence cognitive function to a greater extent than FA values. We speculate that the magnetic susceptibility of the caudate nucleus may be a better MRI-based biomarker reflecting cognitive impairment of patients with AD. Biochemical studies of postmortem brain tissue have proven that the globus pallidus, putamen, red nucleus, and substantia nigra have the highest iron content (Griffiths and Crossman, 1993; Cornett et al., 1998; Hebbrecht et al., 1999). The basal ganglia is a major iron-accumulating brain area, especially with pathological degenerative changes, and iron accumulation in the brain of patients with AD is relevant to senile plaques and neurofibrillary tangles (Lovell et al., 1998; Masaldan et al., 2019). Iron-mediated events, such as iron leading to hyper-phosphorylation and accumulation of tau (Chan and Shea, 2006), may aggravate neurodegeneration and functional impairment, which are more complicated than iron-related oxidative injury (Masaldan et al., 2019).

Another major discovery was that the AxD values of the right SCR, right ACR, right ALIC, and right PLIC were positively correlated with the susceptibility values of the right caudate nucleus. The study has shown that disease-associated demyelination can reduce FA but does not affect AxD (Rovaris et al., 2005), indirectly providing the possibility that iron may affect AxD. In addition, some scholars have found that increased magnetic susceptibility value is associated with white matter damage (Morey et al., 2015). The myelin’s compact layers of the lamellae are held together with proteins, but they are vulnerable to destruction from reactive oxidative substances, such as iron-associated oxide and lipid peroxidation due to secondary degeneration (Baumann and Pham-Dinh, 2001), reflecting that iron does affect white matter function to a certain extent. The increased iron content and iron oxide in the caudate nucleus may cause damage to the axons and myelin, which may lead to the diffusion dysfunction of water molecules. Increased susceptibility values in specific brain regions due to excessive iron and demyelination of the white matter may indicate impaired cognitive function (Haacke et al., 2005; Carmeli et al., 2014). The explanation for asymmetry is that the right hemisphere is the important functional hemisphere and the structural and functional abnormalities in the right hemisphere may lead to earlier clinical manifestations. Therefore, at the mild stage, it may be easy to detect patients with AD who have brain structural changes and functional impairment (Ding et al., 2008).

This study found that the susceptibility values of the caudate nucleus contribute more to cognitive decline than the FA values in patients with AD and explored the relationship between diffusion function of the white matter and magnetic susceptibility of the basal ganglia. The findings provide new notions that the changes of susceptibility values due to excessive iron in the basal ganglia have correlations with the alterations of diffusion properties of the white matter in patients with AD. Our results found that the changes of susceptibility values in the right caudate nucleus were correlated with the alterations of AxD of the white matter in patients with AD, suggesting that excessive iron accumulation may affect the diffusion function of the white matter.

## Limitations

This study has some limitations. Firstly, we made TBSS analysis based on whole brain instead of the ROI-based approach, which resulted in existing multiple fiber bundles in a cluster, so the latter can better explore and distinguish the specific white matter fiber associated with cognitive function. We employed two widely recognized brain atlases and had access to relevant documents to improve the accuracy of identifying fiber bundles. Secondly, the number of subjects is not enough, which may reduce the statistical effect of group analysis. In the future, our research will enlarge the number of participants to reveal the relationships between diffusion properties, magnetic susceptibility values, and cognitive function. Thirdly, we targeted only iron-rich basal ganglia and adopted simple but inaccurate manual segmentation method. Two observers separately drew ROIs and obtain the mean values to minimize inaccuracy. Other regions, such as red nucleus, substantia nigra, and hippocampus, are not in the scope of this study. They may be worthy of our follow-up research. Finally, we use the most common iron deposition as the main reason to illustrate the increased susceptibility values without considering other factors. In view of the anatomical complexity of the white matter fibers and the complexity of the biochemical and pathophysiological mechanisms in AD, further research is needed in the future.

## Conclusion

In conclusion, our research found changes in diffusion properties and magnetic susceptibility related to AD. Diffusion properties of the white matter and magnetic susceptibility of the caudate nucleus were correlated with cognitive function, and the latter has a greater impact on cognitive decline. The susceptibility values of the caudate nucleus may be a better MRI-based biomarker of the cognitive impairment of AD. We also concluded that excessive iron accumulation may affect the diffusion function of the white matter.

## Data Availability Statement

The original contributions presented in the study are included in the article/Supplementary Material, further inquiries can be directed to the corresponding author/s.

## Ethics Statement

The studies involving human participants were reviewed and approved by the ethics committee of China-Japan Friendship Hospital. The patients/participants provided their written informed consent to participate in this study. Written informed consent was obtained from the individual(s) for the publication of any potentially identifiable images or data included in this article.

## Author Contributions

XL, LD, BZ, and ZZ analyzed and explained the data and drafted and revised the manuscript. LD and GM designed the study. WG, BL, and JL searched and managed the literature. YC, YW, and HY collected data. All authors approved the final manuscript.

## Conflict of Interest

The authors declare that the research was conducted in the absence of any commercial or financial relationships that could be construed as a potential conflict of interest.

## Footnotes

1 https://fsl.fmrib.ox.ac.uk/fsl/fslwiki/FslInstallation

2 https://people.eecs.berkeley.edu/~chunlei.liu/software.html
