# Supplementary material for: Alterations and Associations Between Magnetic Susceptibility of the Basal Ganglia and Diffusion Properties in Alzheimer’s Disease
Source: Front Neurosci. 2021 Feb 16;15:616163. doi: 10.3389/fnins.2021.616163 (PMC7921325; doi:10.3389/fnins.2021.616163)
Supplement: Supplementary file 1 [file Data_Sheet_1.pdf]

Supplementary Material

Supplementary Table 1. Interobserver agreement for the ROI measurements.

ROIs	Left hemisphere ICC (95% CI)	Right hemisphere ICC (95% CI)
Caudate nucleus	0.991 (0.984-0.995)	0.991 (0.985-0.995)
Putamen	0.971 (0.949-0.984)	0.967 (0.943-0.981)
Globus pallidus	0.969 (0.945-0.982)	0.894 (0.819-0.939)

ROI, region of interest; ICC, intraclass correlation coefficient; CI, confidence interval.

Supplementary Table 2. Clusters show differences in RD values.

Cluster ID	Cluster size	P	MNI coordinate			Tracts in clusters
			x	y	z	
AD > HC						
Cluster 1	10	0.001	68	134	87	Anterior limb of internal capsule (R)
Cluster 2	183	0.011	77	147	92	Anterior corona radiata (R)
						Genu of corpus callosum

The MNI coordinate refers to the coordinate with the largest 1-P value. RD, radial diffusivity; AD, Alzheimer's disease; HC, healthy controls; MNI, Montreal Neurological Institute; R, right.

Supplementary Table 3. Clusters show differences in FA values.

Cluster ID	Cluster size	P	MNI coordinate			Tracts in clusters
			x	y	z	
AD < HC						
Cluster 1	318	0.002	76	156	90	Anterior corona radiata (R)
						Genu of corpus callosum

The MNI coordinate refers to the coordinate with the largest 1-P value. FA, fractional anisotropy; AD, Alzheimer's disease; HC, healthy controls; MNI, Montreal Neurological Institute; R, right.

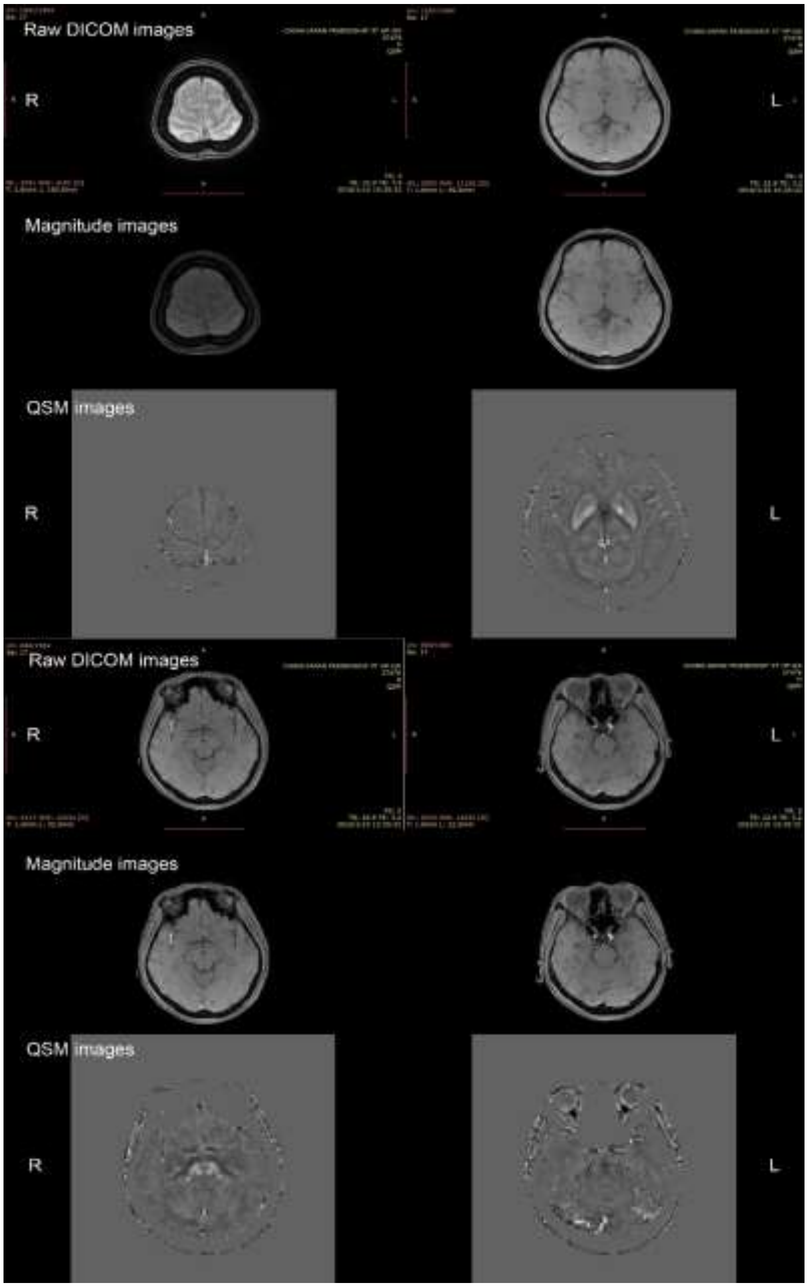

Supplementary Figure 1. Image of the four slices of the same patient's brain. In the figure, the raw and unprocessed DICOM images, the magnitude images, and the QSM images post-processed by STI Suite software were divided into four columns, which came from four brain levels. Take the three images in the first three rows, the second column as an example, they are all at the same basal ganglia level, which are the raw DICOM image, magnitude image, and QSM image, respectively.

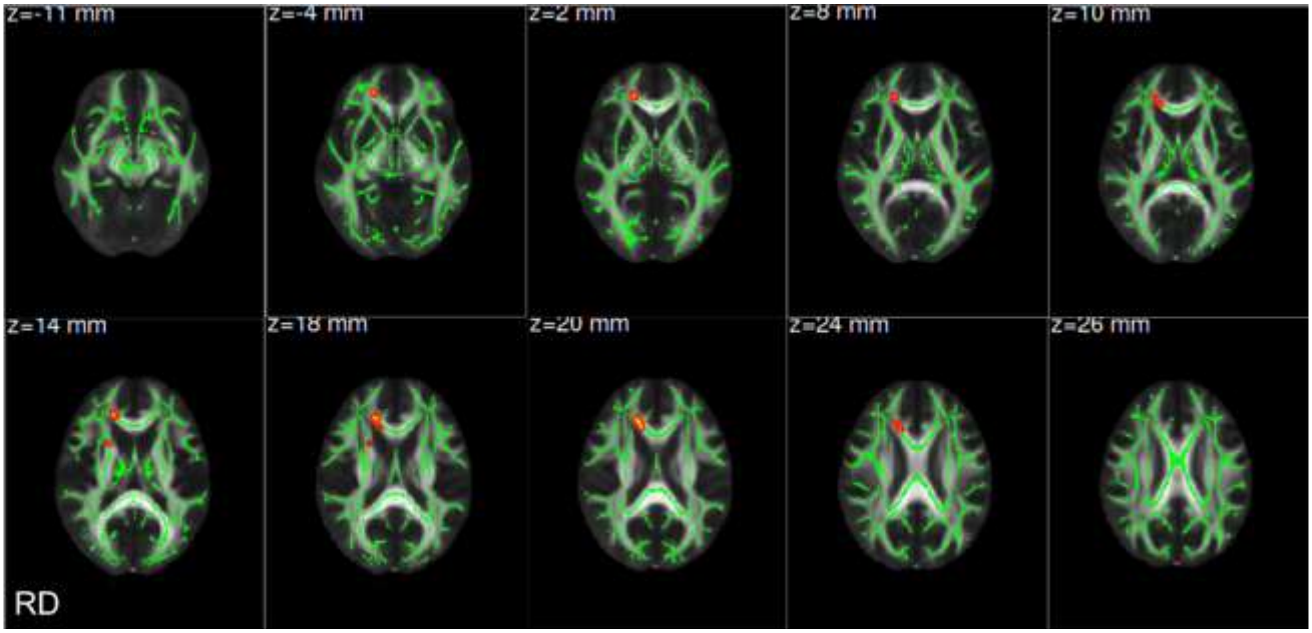

Supplementary Figure 2. Clusters showing radial diffusivity (RD) differences between patients with Alzheimer's disease (AD) and healthy controls (HC). The red clusters showed increased RD values in the AD group compared to the HC group (AD > HC; uncorrected $p < 0.05$, FWE corrected $p > 0.05$). FWE, family wise error.

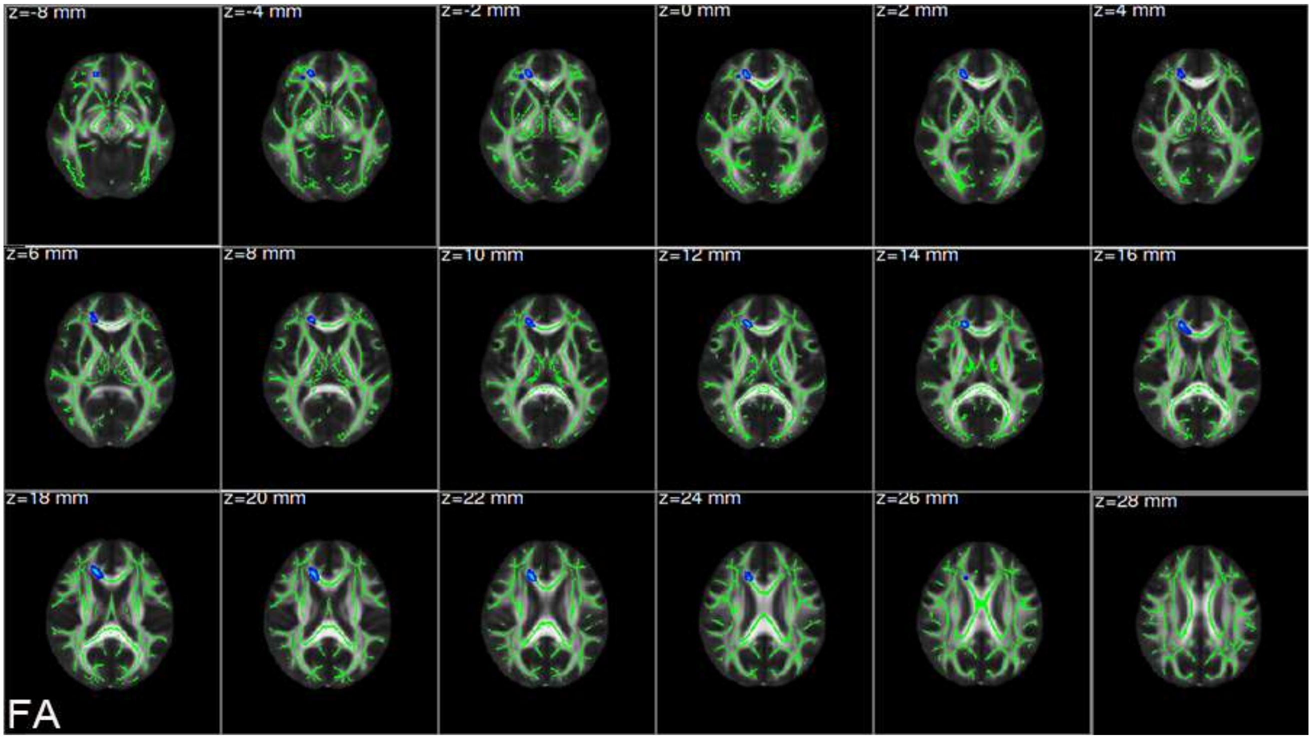

Supplementary Figure 3. Clusters showing fractional anisotropy (FA) differences between patients with Alzheimer's disease (AD) and healthy controls (HC). The blue clusters were attached to the axial images of the mean FA skeleton (green), showing decreased FA values in the AD group compared to the HC group (AD < HC; uncorrected $p = 0.002$, FWE corrected $p > 0.05$). FWE, family wise error.
